# Supplementary material for: Generation of Rhesus Macaque Embryos with Expanded CAG Trinucleotide Repeats in the Huntingtin Gene
Source: Cells. 2024 May 13;13(10):829. doi: 10.3390/cells13100829 (PMC11119628; doi:10.3390/cells13100829)
Supplement: Supplementary file 1 [file cells-13-00829-s001.zip › cells-2877601-supplementary.pdf]

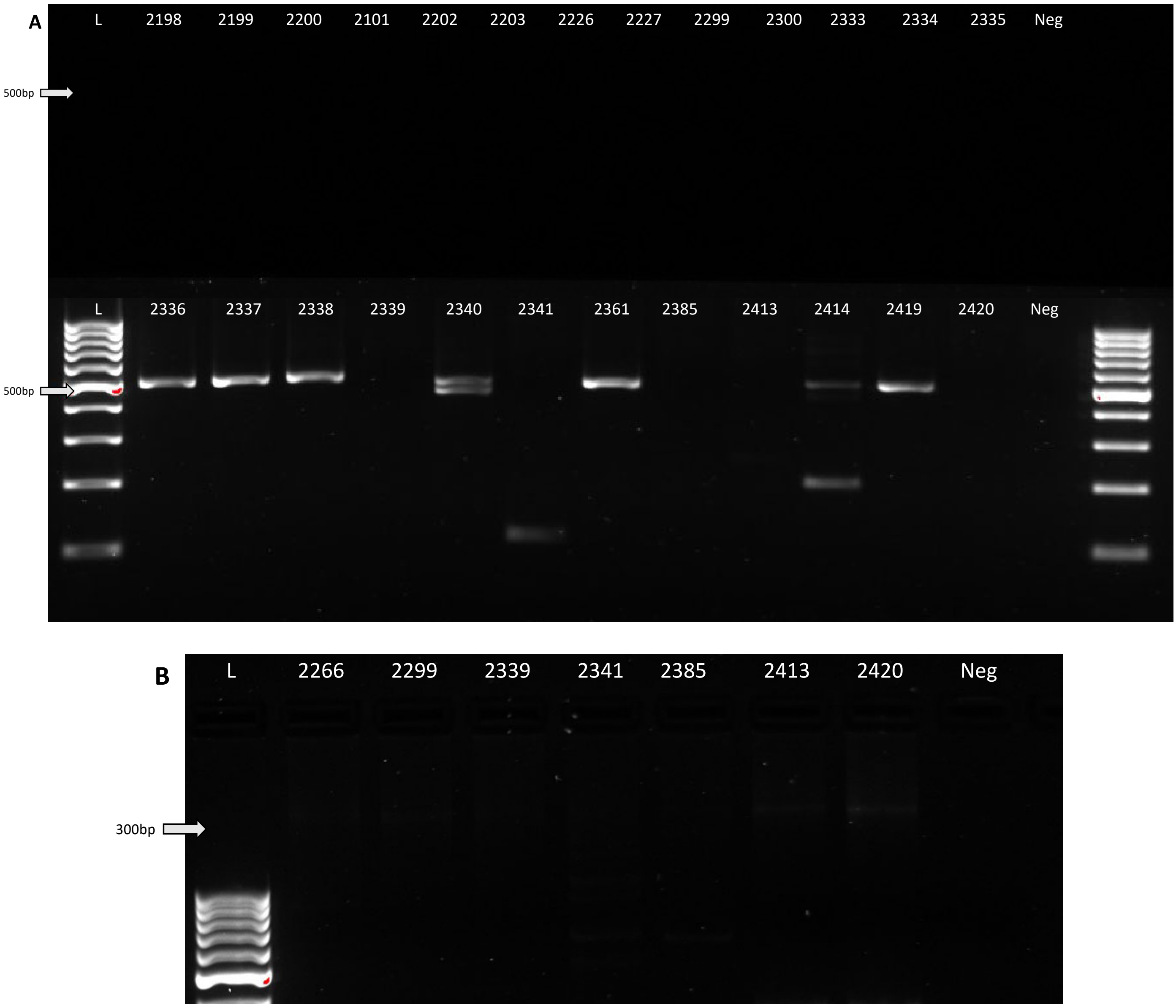

Supplementary Figure S1. Verification of ssDNA integration in TE Biopsy samples. (A) A total of 25 TE biopsy samples from blastocysts underwent amplification of the flanking region of the *HTT* gene. The individual embryo identification numbers are listed on the top of each lane. Among these, 18 samples displayed PCR amplicons, while 7 samples did not yield PCR amplicons. Within the 18 samples, 2 exhibited random indel mutations (#2340 and #2414). The negative control sample (Neg) includes a PCR reaction without genomic DNA. (B) *MYO7A* genes were amplified from 6 TE samples (Supplementary Table S1). The PCR results revealed that out of these, 5 failed to amplify the *MYO7A* gene, implying potential WGA failure. In the case of two samples, *MYO7A* gene amplicons were detected that indicated large deletions in the *HTT* gene.

A

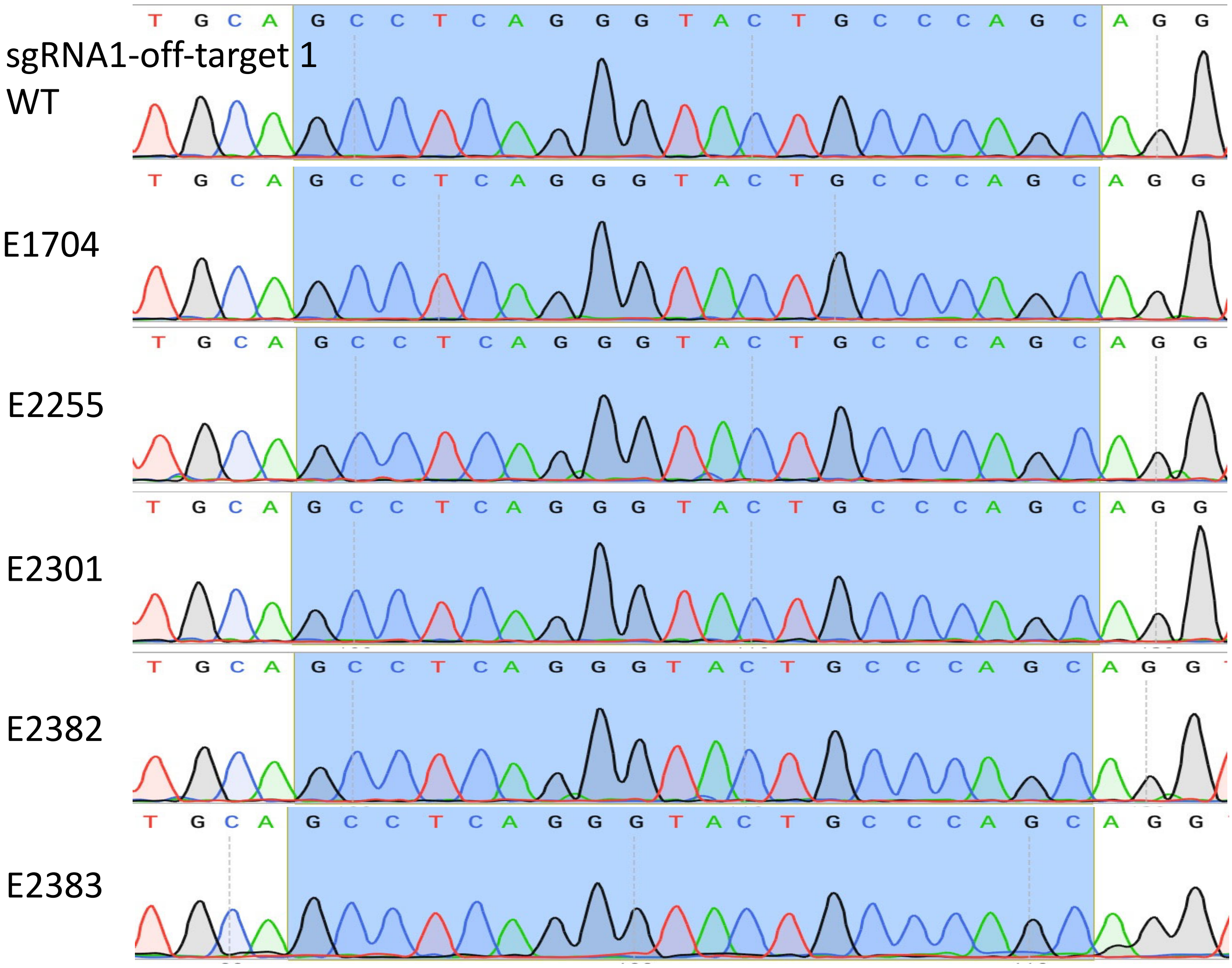

B

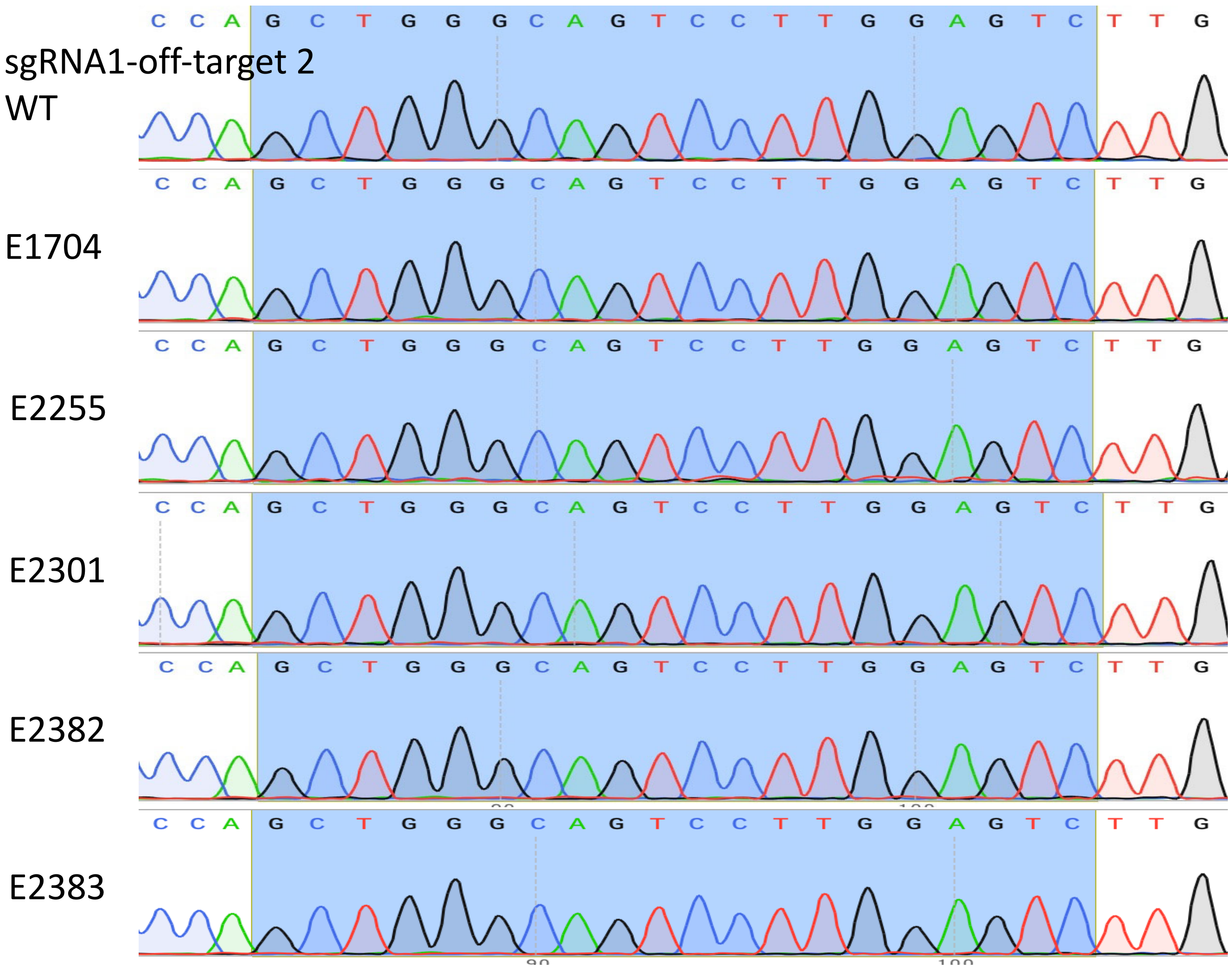
sgRNA1-off-target 2
WT
C C A G C T G G G C A G T C C T T G G A G T C T T G
E1704
C C A G C T G G G C A G T C C T T G G A G T C T T G
E2255
C C A G C T G G G C A G T C C T T G G A G T C T T G
E2301
C C A G C T G G G C A G T C C T T G G A G T C T T G
E2382
C C A G C T G G G C A G T C C T T G G A G T C T T G
E2383
C C A G C T G G G C A G T C C T T G G A G T C T T G

C

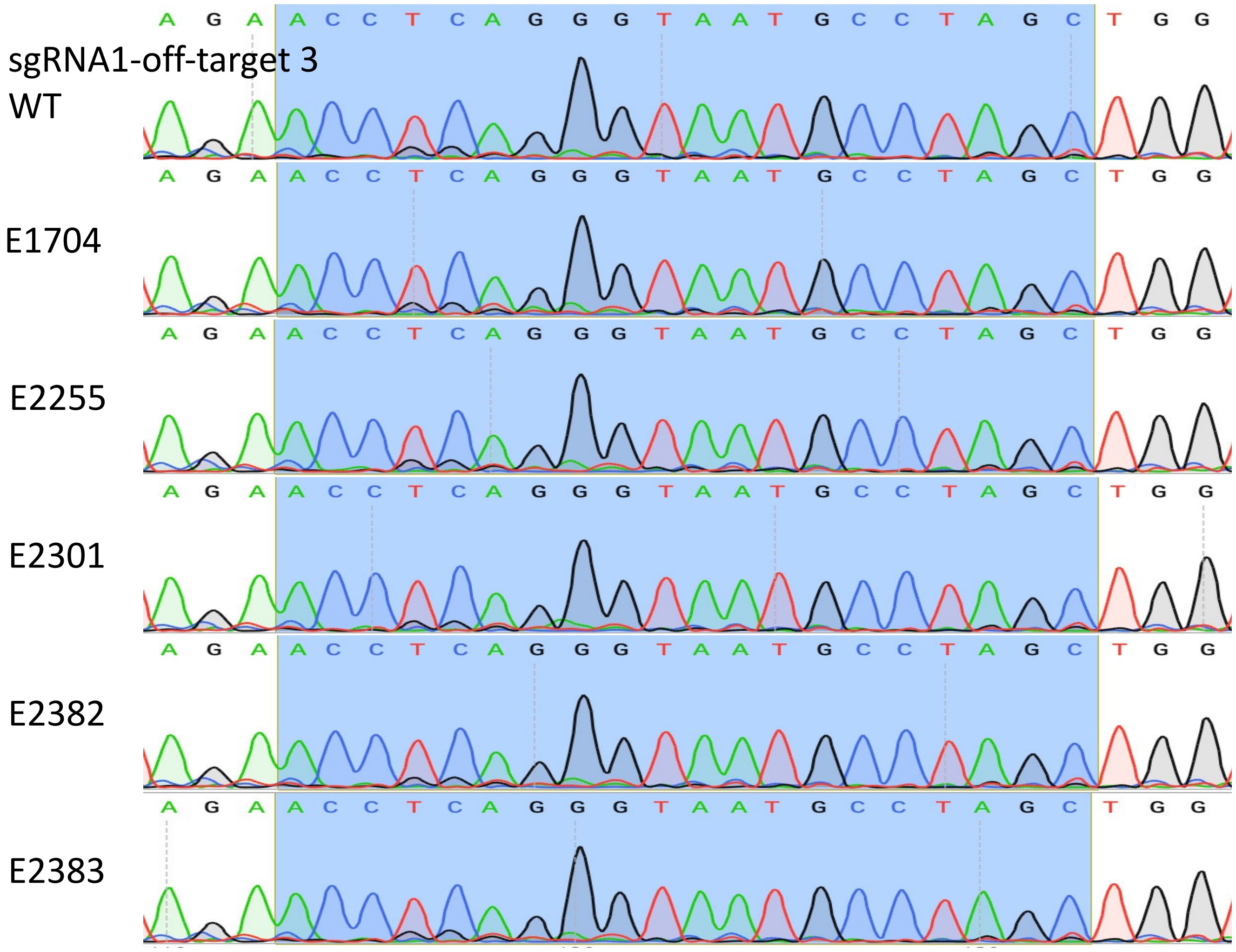
sgRNA1-off-target 3
WT
E1704
E2255
E2301
E2382
E2383
A G A A C C T C A G G G T A A T G C C T A G C T G G

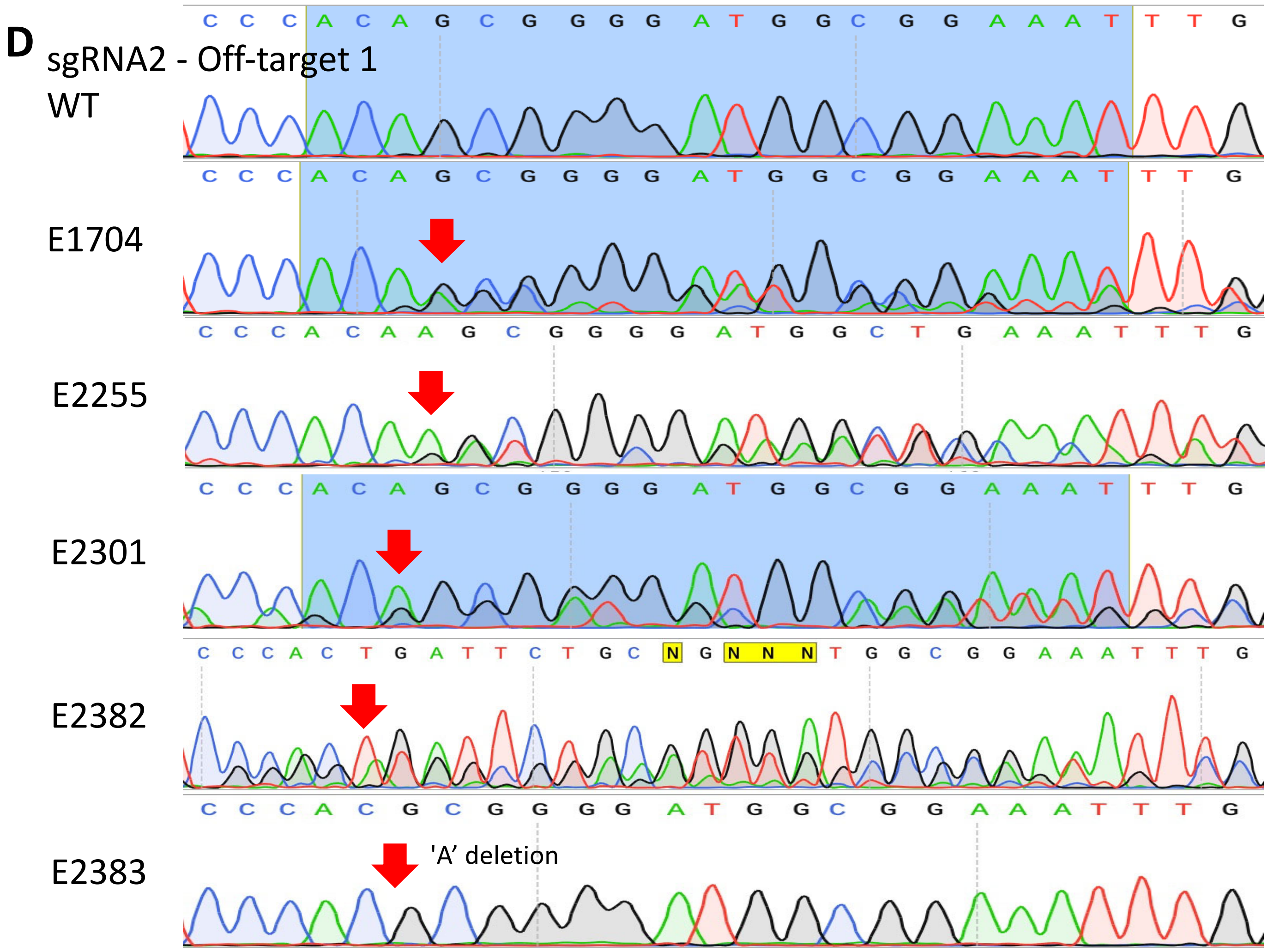
D
sgRNA2 - Off-target 1
WT
C C C A C A G C G G G G A T G G C G G A A A T T T G
E1704
C C C A C A G C G G G G A T G G C G G A A A T T T G
E2255
C C C A C A A G C G G G G A T G G C T G A A A T T T G
E2301
C C C A C A G C G G G G A T G G C G G A A A T T G
E2382
C C C A C T G A T T C T G C N G N N N T G G C G G A A A T T T G
E2383
C C C A C G C G G G G A T G G C G G A A A T T T G
'A' deletion

E

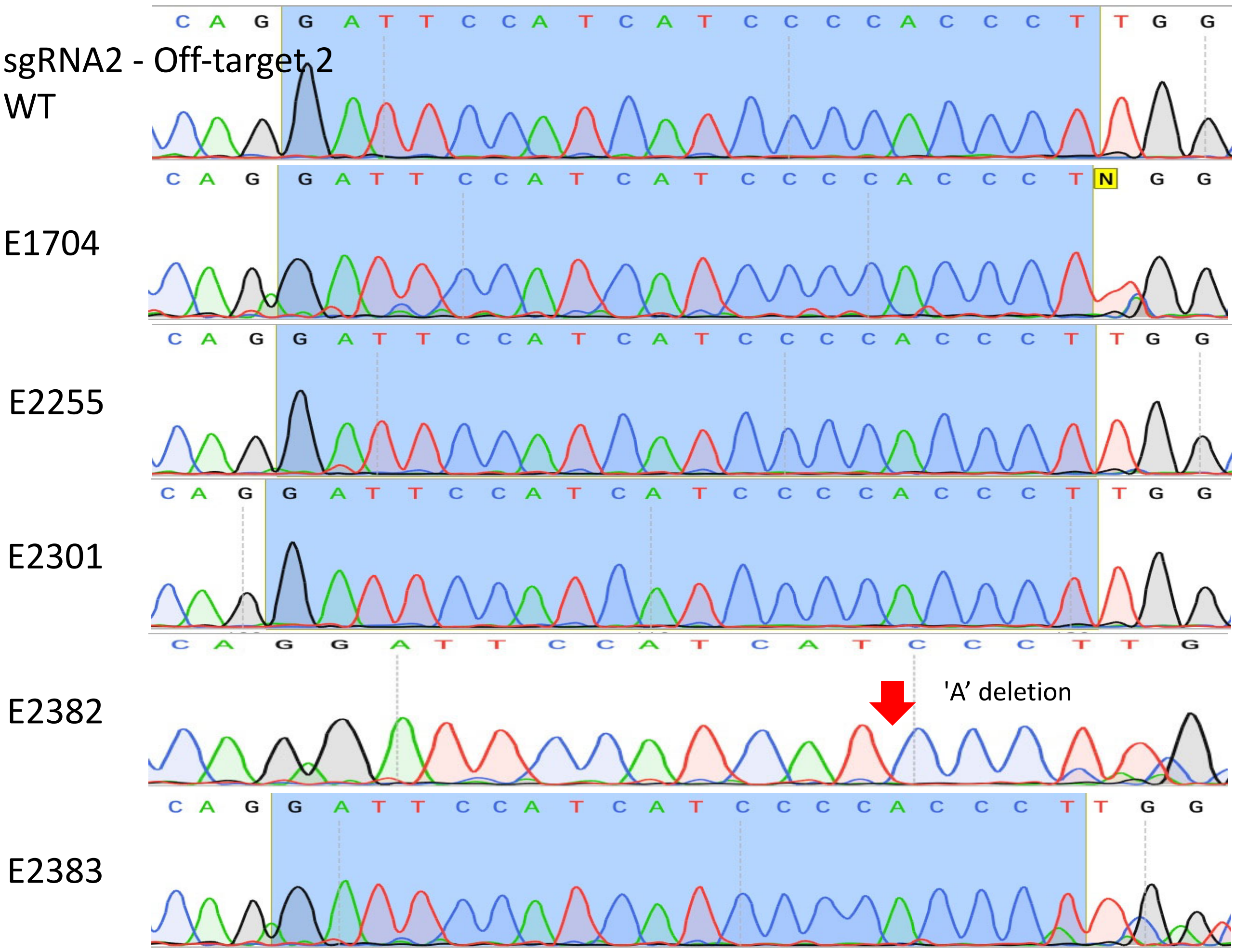
sgRNA2 - Off-target 2
WT
C A G G A T T C C A T C A T C C C C A C C C T T G G
E1704
C A G G A T T C C A T C A T C C C C A C C C T N G G
E2255
C A G G A T T C C A T C A T C C C C A C C C T T G G
E2301
C A G G A T T C C A T C A T C C C C A C C C T T G G
E2382
C A G G A T T C C A T C A T C C C T T G
'A' deletion
E2383
C A G G A T T C C A T C A T C C C C A C C C T T G G

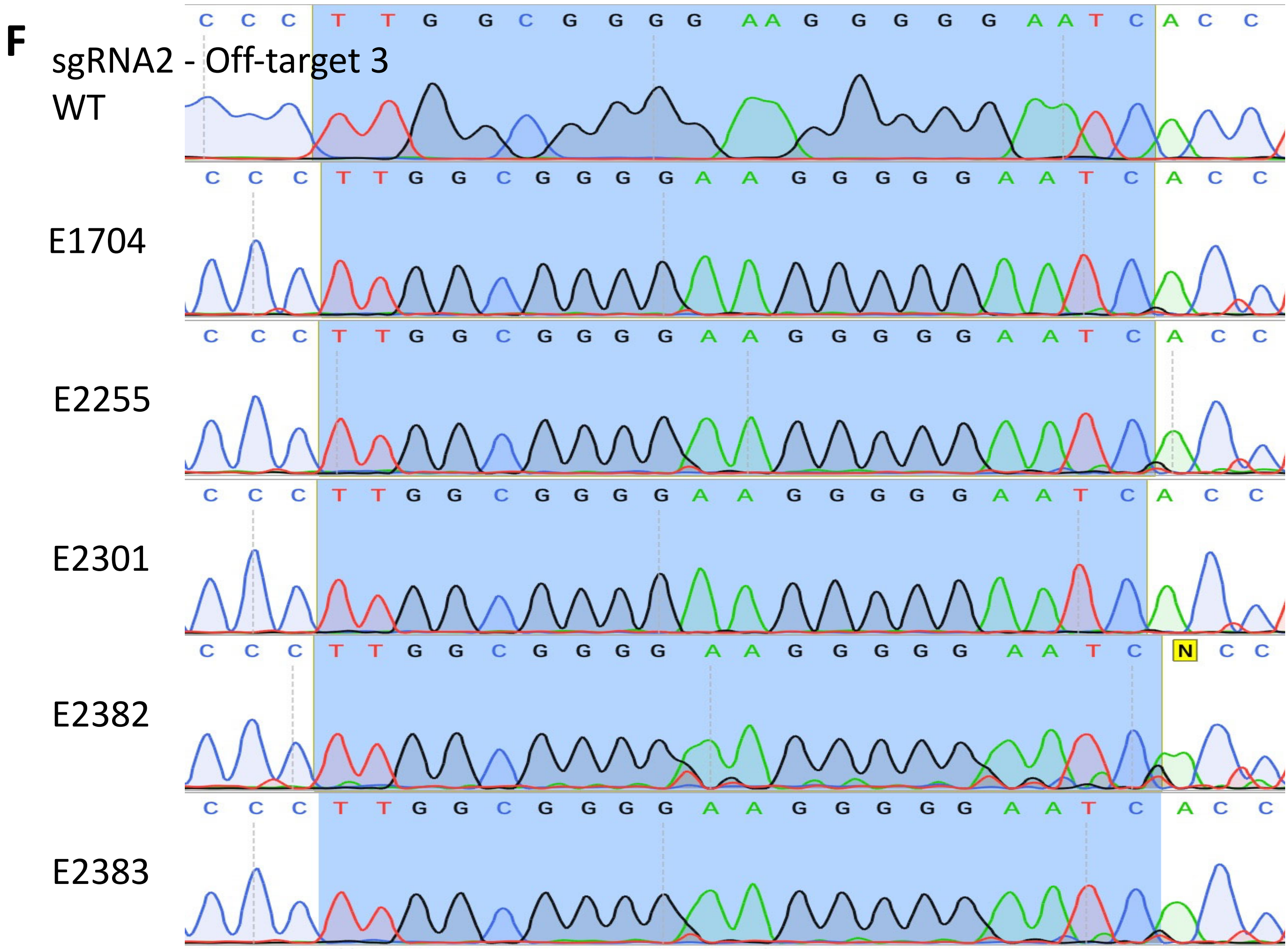
F
sgRNA2 - Off-target 3
WT
E1704
E2255
E2301
E2382
E2383

G

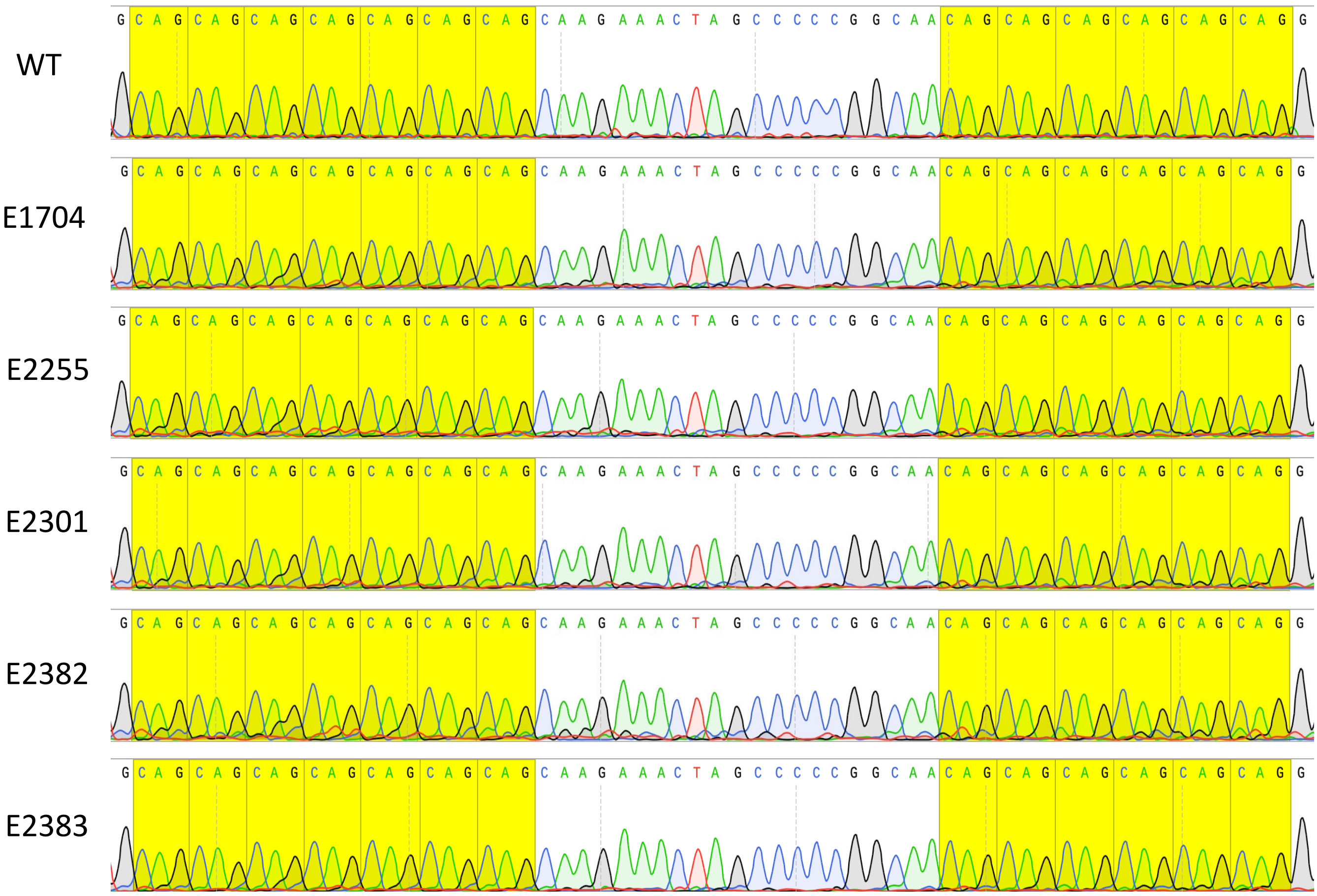

Supplementary Figure S2. Off-target analysis of TE biopsy samples from cryopreserved embryos. A total 6 off-target sites and exon 1 of androgen receptor were amplified by PCR and analyzed by Sanger sequencing. (A-C) Top three scoring of off-target sites for sgRNA1. (D-F) Top three scoring of off-target sites for sgRNA2. Off-target events were detected from off-target site 1 and site 2. Red arrows were indicated mutations. Blue highlights were wild-type sequence. (G) Androgen receptor exon 1 has CAG repeats (yellow highlight) and there was no integration of ssDNA on androgen receptor exon 1.

Supplementary Table S1. Primer information and ssDNA template sequence.

Primer name	Primer sequence (5'-3')	PCR product size
HTT forward	CCAAGATGGACGGCCGCTT	WT – 508bp ssDNA integration – 710bp
HTT reverse	CTCATTGGCTCTGGGTTGCTGGGTCAC	
MYO7A forward	CACGACTCCAAAGCCAGGACTCTTC	351bp
MYO7A reverse	ACAGCTGGAGCTCTAGGTCCTAAGC	
OT-sgRNA1-1 forward	GGGCAACATAGTGAGACCTTGCCTCTATTATAAAG	327bp
OT-sgRNA1-1 reverse	CATGTTAAAAATGCAGGGCCTTCAAGAGATTTTC	
OT-sgRNA1-2 forward	GTCCAGTGTTGATTCCCTTAGAGGAGAAAC	387bp
OT-sgRNA1-2 reverse	AGCAGCTATCAGAAGCGGACTCCTC	
OT-sgRNA1-3 forward	CTCCATTCTTGTTATTGGCCAGAACTTGGTC	349bp
OT-sgRNA1-3 reverse	GGGCTAATTTTTTTATATTTTGTAGAGACGGGACCTTG	
OT-sgRNA2-1 forward	CTACGGGAAGAACGCATTAGTCACTTTGC	262bp

OT-sgRNA2-1 reverse	GCTGATCTTTGTCCACCCAAAAAGGTGTC	
OT-sgRNA2-2 forward	CAAACCAGGAAGTGGTCTTAATTATCACAATGTAGATTAGC	254bp
OT-sgRNA2-2 reverse	GGACAGAGCCTGAGATGAGACTCTAAGAAAAAG	
OT-sgRNA2-3 forward	TGTACATTGGGGTGTTCACAGCACC	254bp
OT-sgRNA2-3 reverse	CTTCTCCTCCACCCTCCTCTCTGAAAC	
AD receptor forward	CAAGACCTACCGAGGAGCTTTCCAGAATC	256bp
AD receptor reverse	CTGTGAAGGCTGCTGTTCCTCATCCAG	
ssDNA template	CATTCATTGCCCCGGTGCTGAGCGGCGCTGCGAGTCGGCCCGAGGCCTCCGGGAC<br>TGCCTTGCCGGGCGGGAGACCGCCATGGCGACCCTGGAAAAGCTGATGAAGGCCTT<br>CGAGTCCCTCAAGTCCTTCCAGCAGCAGCAGCAGCAGCAGCAGCAGCAGCAGCAGC<br>AGCAGCAGCAGCAGCAGCAGCAGCAGCAGCAGCAGCAGCAGCAGCAGCAGCAGC<br>AGCAGCAGCAGCAGCAGCAGCAGCAGCAGCAGCAGCAGCAGCAGCAGCAGCAGC<br>AGCAGCAGCAGCAGCAGCAGCAGCAGCAGCAGCAGCAGCAGCAGCAGCAGCAGC<br>AGCAGCAGCAGCAGCAGCAGCAGCAGCAGCAACAGCCGCCACCGCCGCCGCCGCC<br>GCCGCCGCCTCCTCAGCTTCCTCAGCCGCCGCCGCAGGCACAGCCGCTGCTGCCTCA<br>GCCGCAGCCGCCCCCGCCGCCGCCCCCGCCGCCACCCGGCCCGGCTGTGGCTGAGG<br>AGCCGCTGCACCGACCGTGAGTTTGGGCCCGCTGCAGCTCCCTGTCCCGGCGGGTC<br>CCAGGCTACGGCGGGGATGGCGGAATCCTGCAGCCTGCGGGCCGGCGACACGAAC<br>CCCCCCGGCCCCGCAG	625bp
